# Supplementary material for: CRISPRi-based screens in iAssembloids to elucidate neuron-glia interactions
Source: bioRxiv. 2024 Aug 12:2023.04.26.538498. Originally published 2023 Apr 27. Preprint. [Version 2] doi: 10.1101/2023.04.26.538498 (PMC10168378; doi:10.1101/2023.04.26.538498)
Supplement: Supplementary file 1 [file NIHPP2023.04.26.538498v2-supplement-8.pdf]

bioRxiv preprint doi: https://doi.org/10.1101/2023.04.26.538498; this version posted August 12, 2024. The copyright holder for this preprint (which was not certified by peer review) is the author/funder, who has granted bioRxiv a license to display the preprint in perpetuity. It is made available under aCC-BY 4.0 International license.

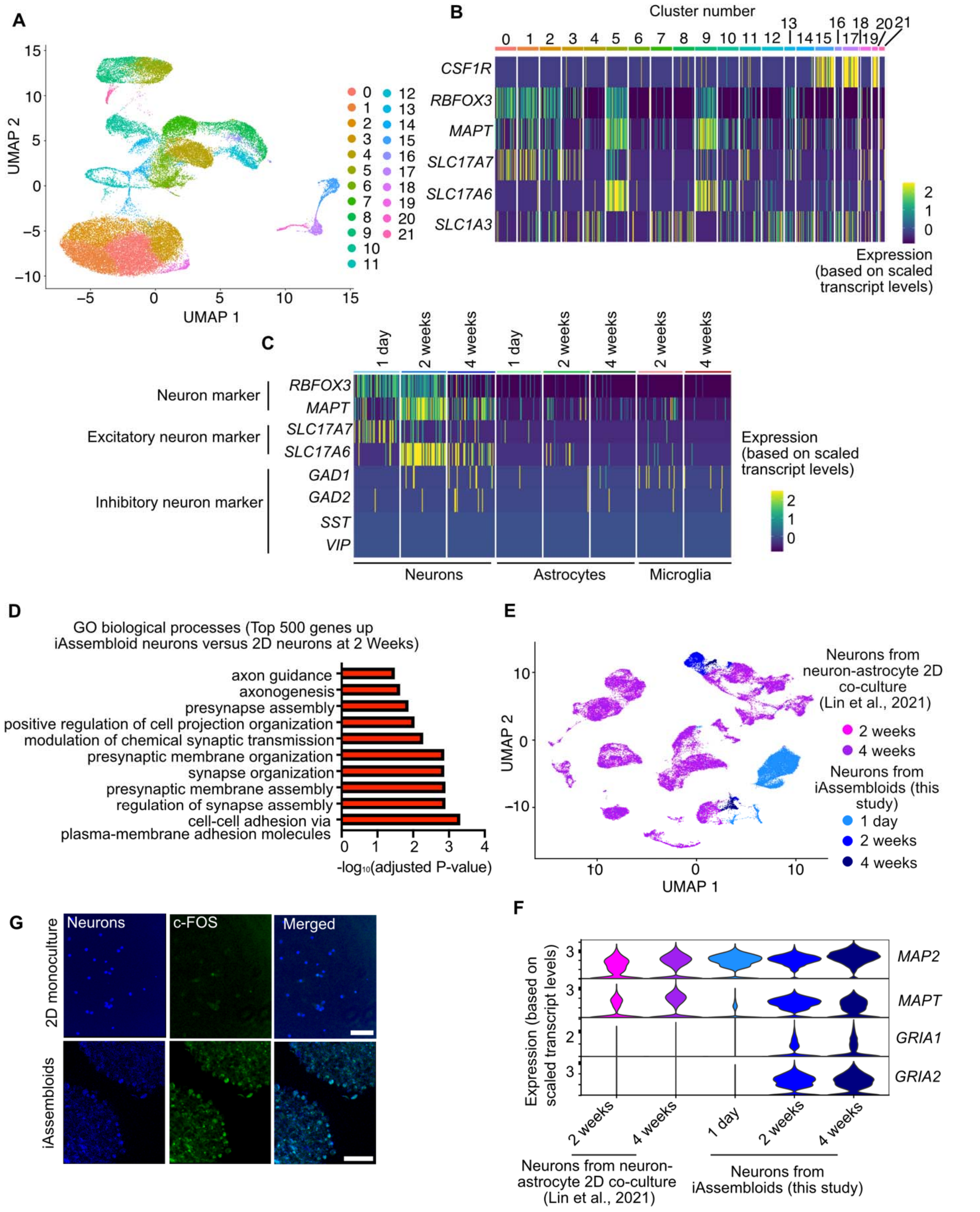


**Figure S1. Single-nucleus RNA sequencing of iAssembloids**

bioRxiv preprint doi: https://doi.org/10.1101/2023.04.26.538498; this version posted August 12, 2024. The copyright holder for this preprint (which was not certified by peer review) is the author/funder, who has granted bioRxiv a license to display the preprint in perpetuity. It is made available under aCC-BY 4.0 International license.

(A) UMAP representation of snRNA sequencing data of iAssembloids. Colors represent clusters assigned through unbiased clustering

(B) Heatmap representing expression of cell type-specific genes for cluster assignment. *CSF1R*: Microglia, *RBFOX3*, *MAPT*, *SLC17A7*, *SLC17A6*: Neurons, *SLC1A3*: Astrocytes

(C) Heatmap representing expression of neuronal subtype-specific markers. *RBFOX3*, *MAPT*: Pan-neuronal markers, *SLC17A7*, *SLC17A6*: excitatory neuron markers, *GAD1*, *GAD2*, *SST*, *VIP*: inhibitory neuron markers.

**(D)** Most significant Gene Ontology Biological Processes enriched in the 500 top genes expressed more highly in neurons in iAssembloids vs. 2D monocultured neurons. Adjusted P values were calculated using the EASE Score, a Modified Fisher Exact P-value and Benjamini-Hochberg method for correction for multiple hypothesis testing.

(E) UMAP representation of integrated dataset from astrocyte and neuron 2D co-culture versus iAssembloids.

(F) Selected differentially expressed genes between neurons from 2D astrocyte-neuron co-culture[20] versus neurons from iAssembloids.

(G) BFP+ neurons (blue) in monoculture and cultured within iAssembloids were stained for c-FOS (green). Scale bars represent 100 µm.

bioRxiv preprint doi: https://doi.org/10.1101/2023.04.26.538498; this version posted August 12, 2024. The copyright holder for this preprint (which was not certified by peer review) is the author/funder, who has granted bioRxiv a license to display the preprint in perpetuity. It is made available under aCC-BY 4.0 International license.

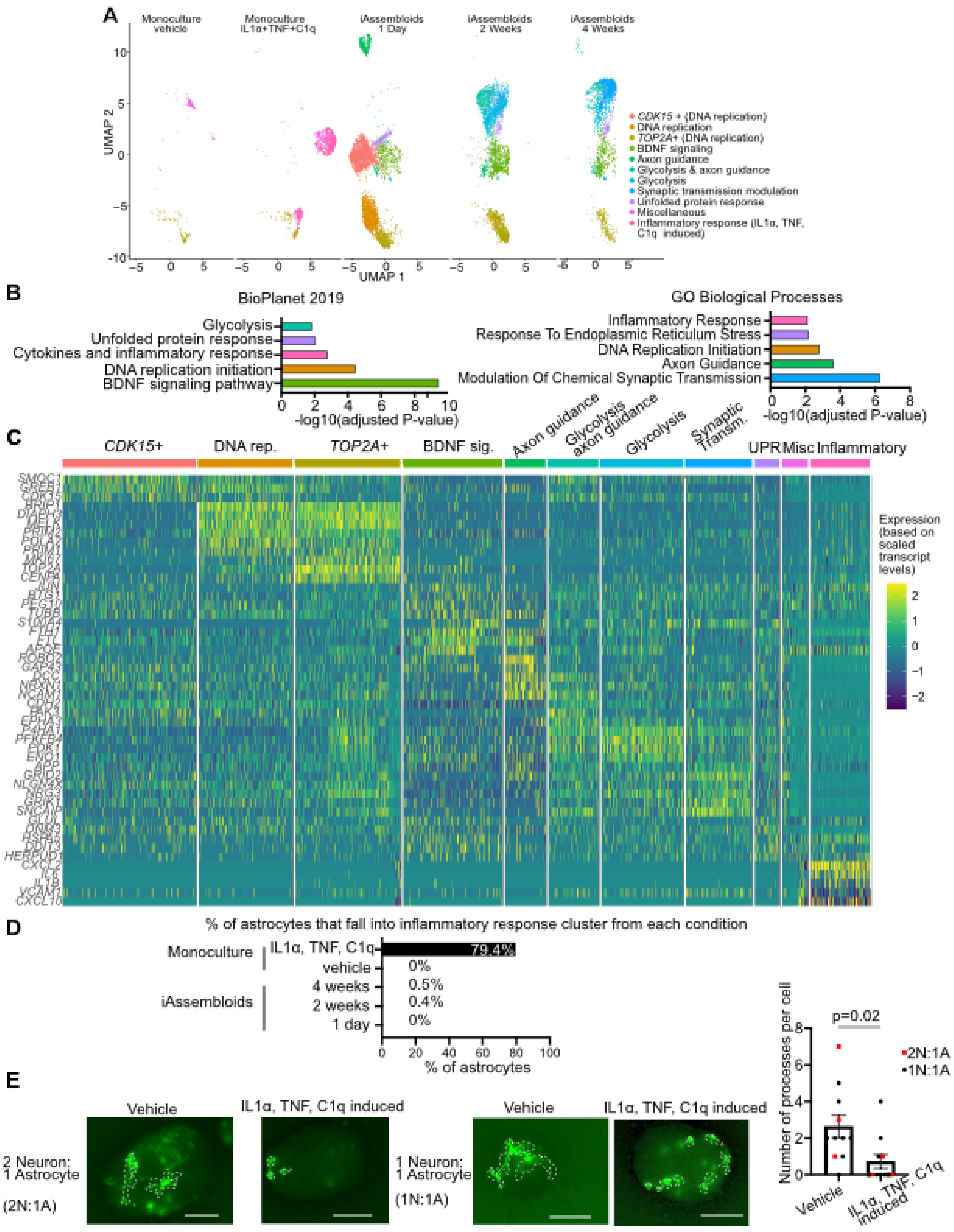

bioRxiv preprint doi: https://doi.org/10.1101/2023.04.26.538498; this version posted August 12, 2024. The copyright holder for this preprint (which was not certified by peer review) is the author/funder, who has granted bioRxiv a license to display the preprint in perpetuity. It is made available under aCC-BY 4.0 International license.

**Figure S2. Astrocytes in iAssembloids express genes associated with neuronal support** (related to Fig. 2)

**(A)** Cells identified as astrocytes from snRNA-seq in iAssembloids (this study) were integrated with data from single cell sequencing of monocultured astrocytes[9]using Seurat's data integration pipeline. A UMAP was generated and split by origin of sample and time point. Cluster names were defined by running defined markers through EnrichR. Clusters include a *CDK15*+ cluster, cells undergoing DNA replication, an immature astrocyte cluster *(TOP2A+)*, cells expressing genes related to BDNF signaling, axon guidance, glycolysis, synaptic transmission modulation, the unfolded protein response, and a cluster that had no specific markers (miscellaneous). The final cluster (IL1α, TNF, C1q induced) is based on previous studies for cytokine-induced markers[9].

**(B)** EnrichR BioPlanet 2019 pathway analysis was used to define clusters. Redundant terms (terms that consist of the same genes) were removed and only the term that had the highest -$\log_{10}$(adjusted p-value) is displayed. E.g. *PRIM2, POLA2, PRIM1* fall into the category of DNA replication initiation and Leading strand biosynthesis, but only DNA replication is displayed here. Colors represent clusters that fell into pathway/gene ontology categories.P represents Fisher's Exact Test. Multiple correction testing is performed with the Benjamini-Hochberg method.

**(C)** Heatmap was generated based on markers defined in A and B.

**(D)** After clustering, the percent of total astrocytes that falls within the "inflammatory response category was plotted

**(E)** Green channel represents astrocytes tagged with GFP sparsely seeded with neurons and non-fluorescent astrocytes. Astrocytes are seed at either a 2 neuron to 1 astroycte or 1 astrocyte to 1 astrocyte ratio. Representative images shown with iAssembloids treated with media (vehicle) and with media plus the triple cytokines (IL1α, TNF, C1q). The number of processes per astrocyte in focus (outlined in white) was manually counted. N=8 cells across 4 iAssembloids for vehicle and 8 cells across 3 iAssembloids for induced.  Standard error of the mean is represented. P-value determined by student's t-test. Scale bar represents 100 micron.

bioRxiv preprint doi: https://doi.org/10.1101/2023.04.26.538498; this version posted August 12, 2024. The copyright holder for this preprint (which was not certified by peer review) is the author/funder, who has granted bioRxiv a license to display the preprint in perpetuity. It is made available under aCC-BY 4.0 International license.

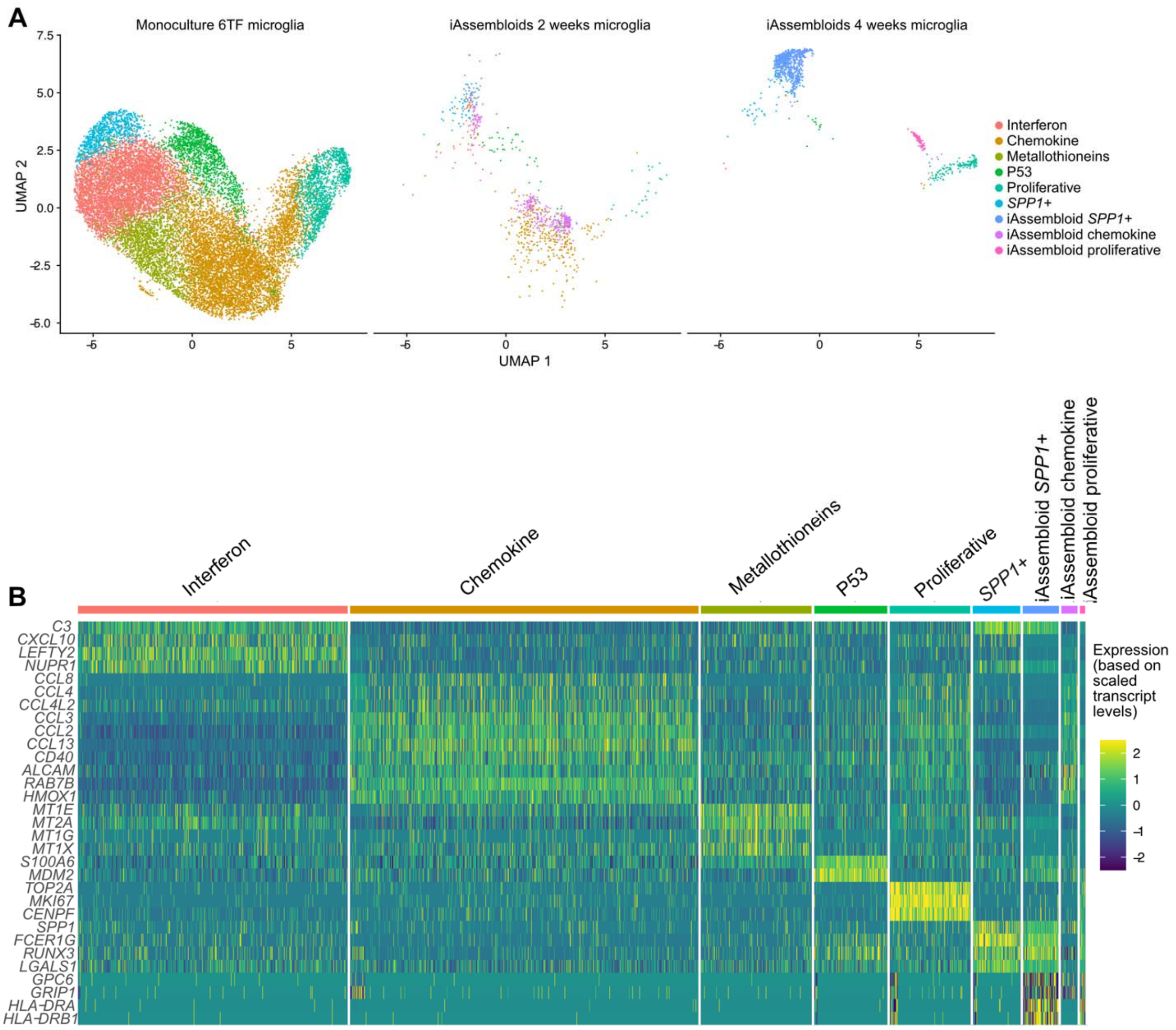


**Figure S3. Microglia within iAssembloids express MHC Class II proteins** (related to Fig. 2)

**(A)** Cells identified as microglia from snRNA-seq in iAssembloids were integrated with single-cell sequencing of microglia[8] using Seurat's data integration pipeline. A UMAP was generated and split by origin of sample and the time point. Clusters include those that fall into the interferon, chemokine, metallothioneins, P54, proliferative and *SPP1+* cluster. iAssembloid microglia specific clusters (iAssembloid *SPP1+,* iAssembloid chemokine, and iAssembloid proliferative are also highlighted). Microglia mainly map to existing clusters. However, cells in iAssembloids uniquely express MHC Class II proteins such as *HLA-DRA* and *HLA-DRB1*.

**(B)** Heatmap was generated based on markers defined in A.

bioRxiv preprint doi: https://doi.org/10.1101/2023.04.26.538498; this version posted August 12, 2024. The copyright holder for this preprint (which was not certified by peer review) is the author/funder, who has granted bioRxiv a license to display the preprint in perpetuity. It is made available under aCC-BY 4.0 International license.

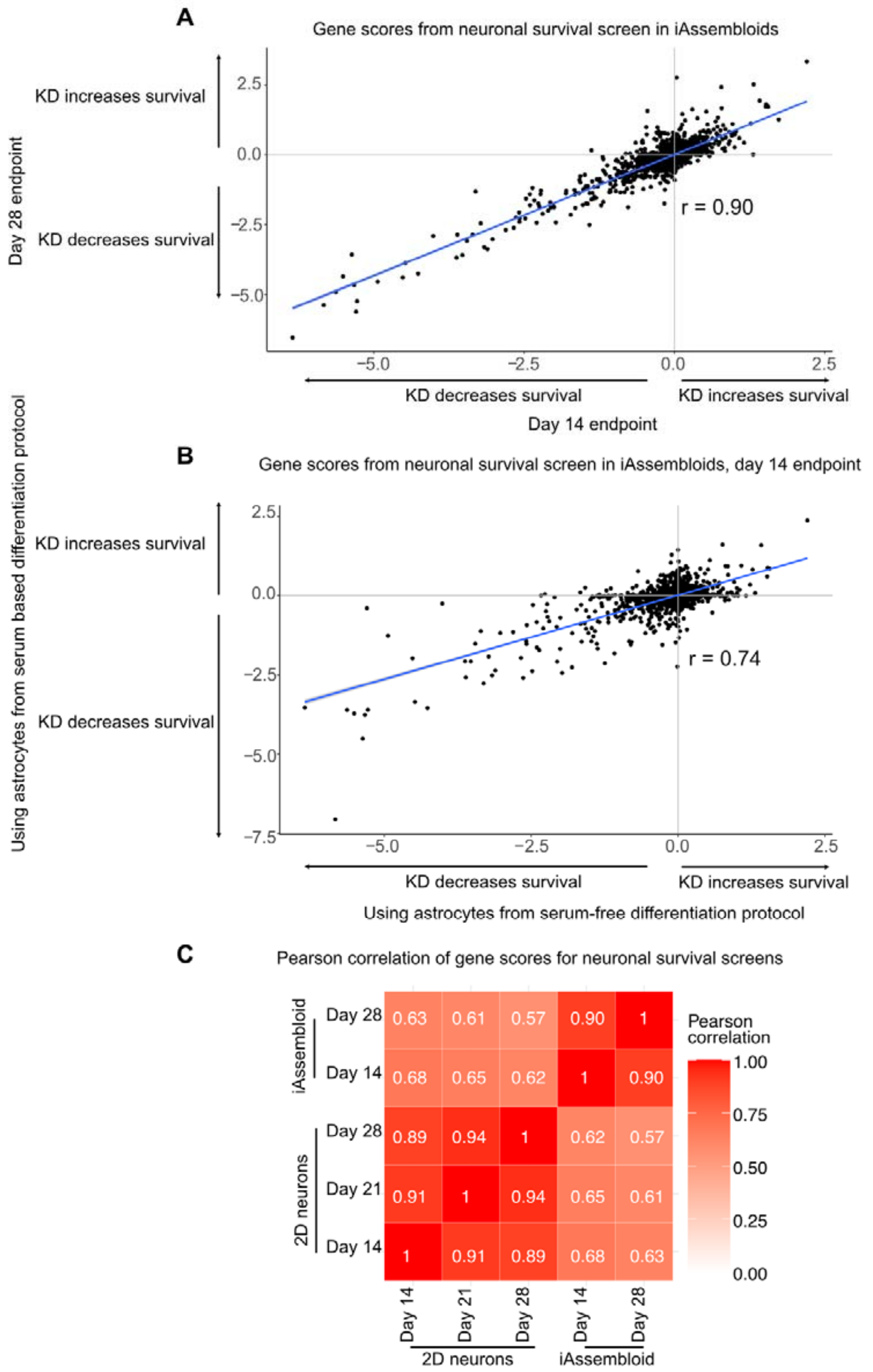


**Figure S4. CRISPRi-based functional genomics screens in iAssembloids are reproducible** (related to Fig. 3)

bioRxiv preprint doi: https://doi.org/10.1101/2023.04.26.538498; this version posted August 12, 2024. The copyright holder for this preprint (which was not certified by peer review) is the author/funder, who has granted bioRxiv a license to display the preprint in perpetuity. It is made available under aCC-BY 4.0 International license.

**(A)** Scatterplot comparing CRISPRi-based neuronal survival screens at 14 days vs. 28 days post seeding into AggreWell plates.

**(B)** Scatterplot comparing CRISPRi-based neuronal survival screens using astrocytes from serum-based differentiation protocol[17,53] vs. the serum-free differentiation protocol[11,12].

**(C)** Correlation heatmap of CRISPRi-based neuronal survival screens in the context of 2D monocultures[6] vs. iAssembloids (this study).

bioRxiv preprint doi: https://doi.org/10.1101/2023.04.26.538498; this version posted August 12, 2024. The copyright holder for this preprint (which was not certified by peer review) is the author/funder, who has granted bioRxiv a license to display the preprint in perpetuity. It is made available under aCC-BY 4.0 International license.

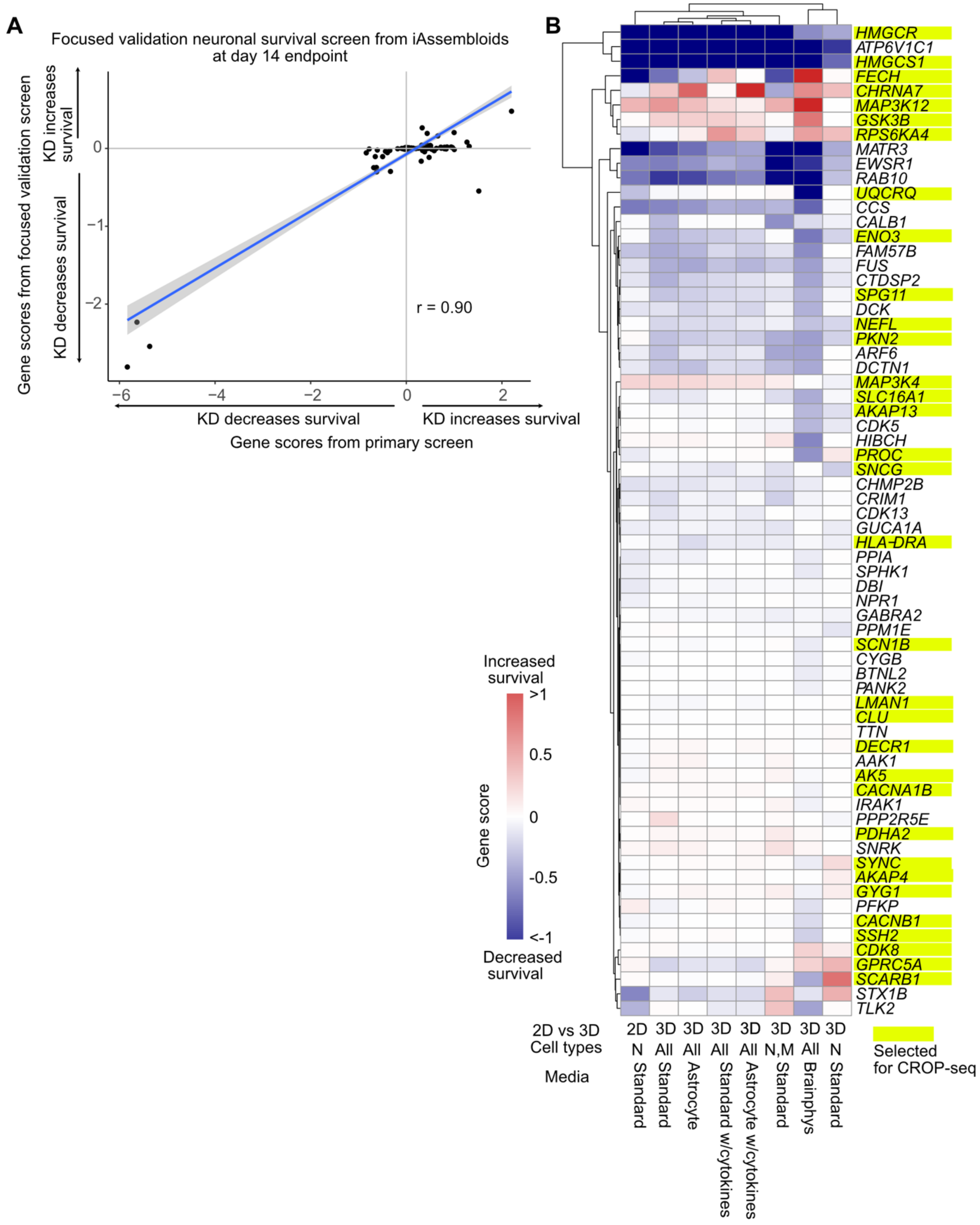


**Figure S5. Focused validation screens for top hits from the primary screen** (related to Fig. 3 and 4)

bioRxiv preprint doi: https://doi.org/10.1101/2023.04.26.538498; this version posted August 12, 2024. The copyright holder for this preprint (which was not certified by peer review) is the author/funder, who has granted bioRxiv a license to display the preprint in perpetuity. It is made available under aCC-BY 4.0 International license.

**(A)** Scatterplot of gene scores from the primary iAssembloid screen hits vs the secondary validation screen. Phenotypes from the secondary screen have a narrower dynamic range but are highly correlated with the primary screen (r = 0.9).

**(B)** Results from secondary screens comparing iAssembloid culture in various media: normal media (composed of 0.5X astrocyte media and 0.5X microglia base media), normal media plus cytokines (our iAssembloid culture condition), astrocyte media, and astrocytes media plus cytokines, as well as 3D cultures with different cell type compositions: neurons only (N) and neurons plus microglia (N,M).

bioRxiv preprint doi: https://doi.org/10.1101/2023.04.26.538498; this version posted August 12, 2024. The copyright holder for this preprint (which was not certified by peer review) is the author/funder, who has granted bioRxiv a license to display the preprint in perpetuity. It is made available under aCC-BY 4.0 International license.

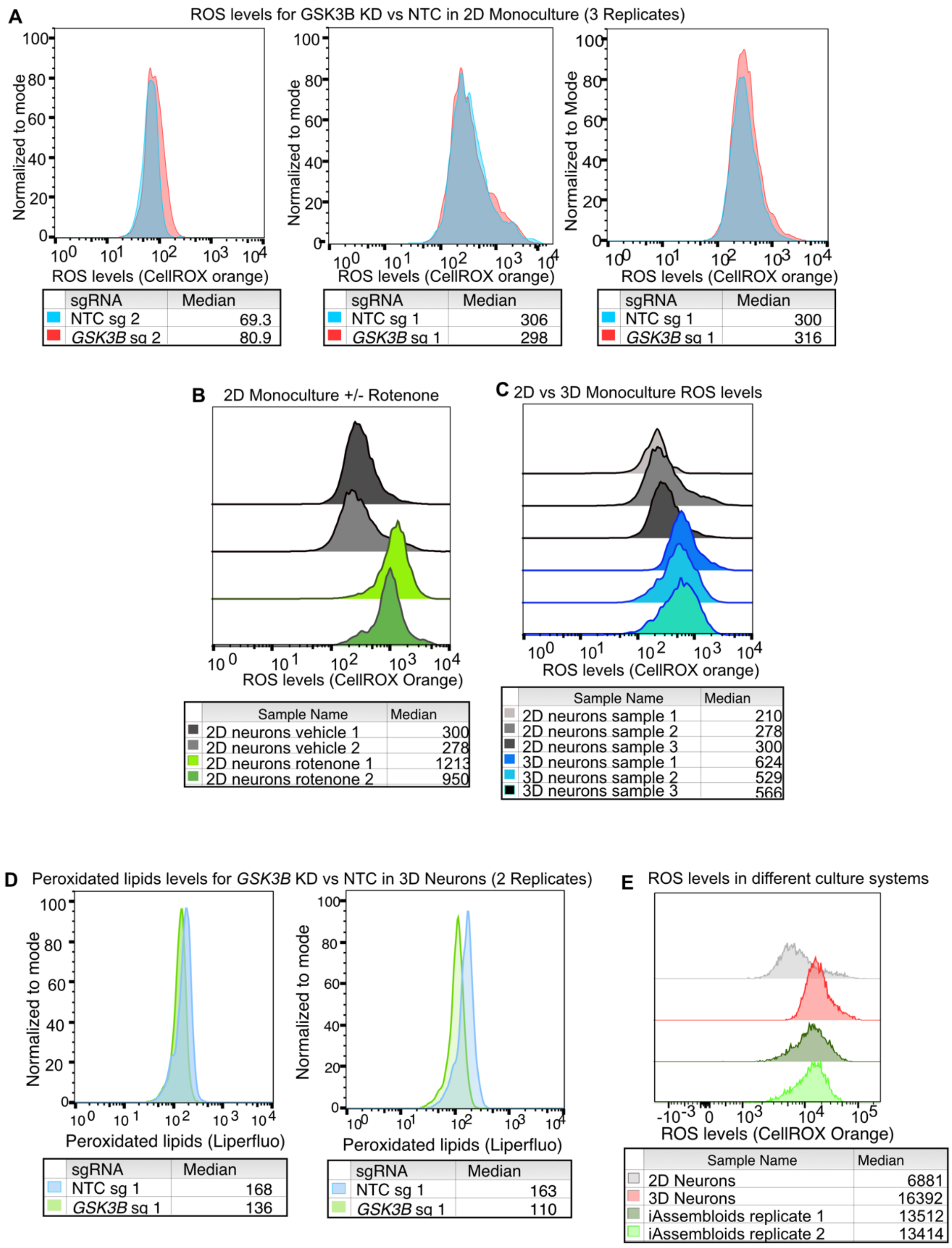


**Figure S6. Representative flow cytometry results for neuronal phenotypes** (related to Fig. 5)

bioRxiv preprint doi: https://doi.org/10.1101/2023.04.26.538498; this version posted August 12, 2024. The copyright holder for this preprint (which was not certified by peer review) is the author/funder, who has granted bioRxiv a license to display the preprint in perpetuity. It is made available under aCC-BY 4.0 International license.

**(A)** Three replicates of 2D monoculture neurons with and without *GSK3B* knockdown stained with CellROX$^{TM}$ orange.

**(B)** Replicates of 2D vs 3D monocultured neurons stained for ROS levels by CellROX$^{TM}$ orange.

**(C)** Replicates of 2D monocultured neurons with and without rotenone treatment stained for ROS levels by CellROX$^{TM}$ orange.

**(D)** Replicates of 3D cultured neurons with and without GSK3B knockdown stained with Liperfluo to detect peroxidated lipid levels

**(E)** Neurons from 3 different culture systems (2D monoculture, 3D monoculture, iAssembloids) stained for ROS levels by CellROX$^{TM}$ orange.

bioRxiv preprint doi: https://doi.org/10.1101/2023.04.26.538498; this version posted August 12, 2024. The copyright holder for this preprint (which was not certified by peer review) is the author/funder, who has granted bioRxiv a license to display the preprint in perpetuity. It is made available under aCC-BY 4.0 International license.

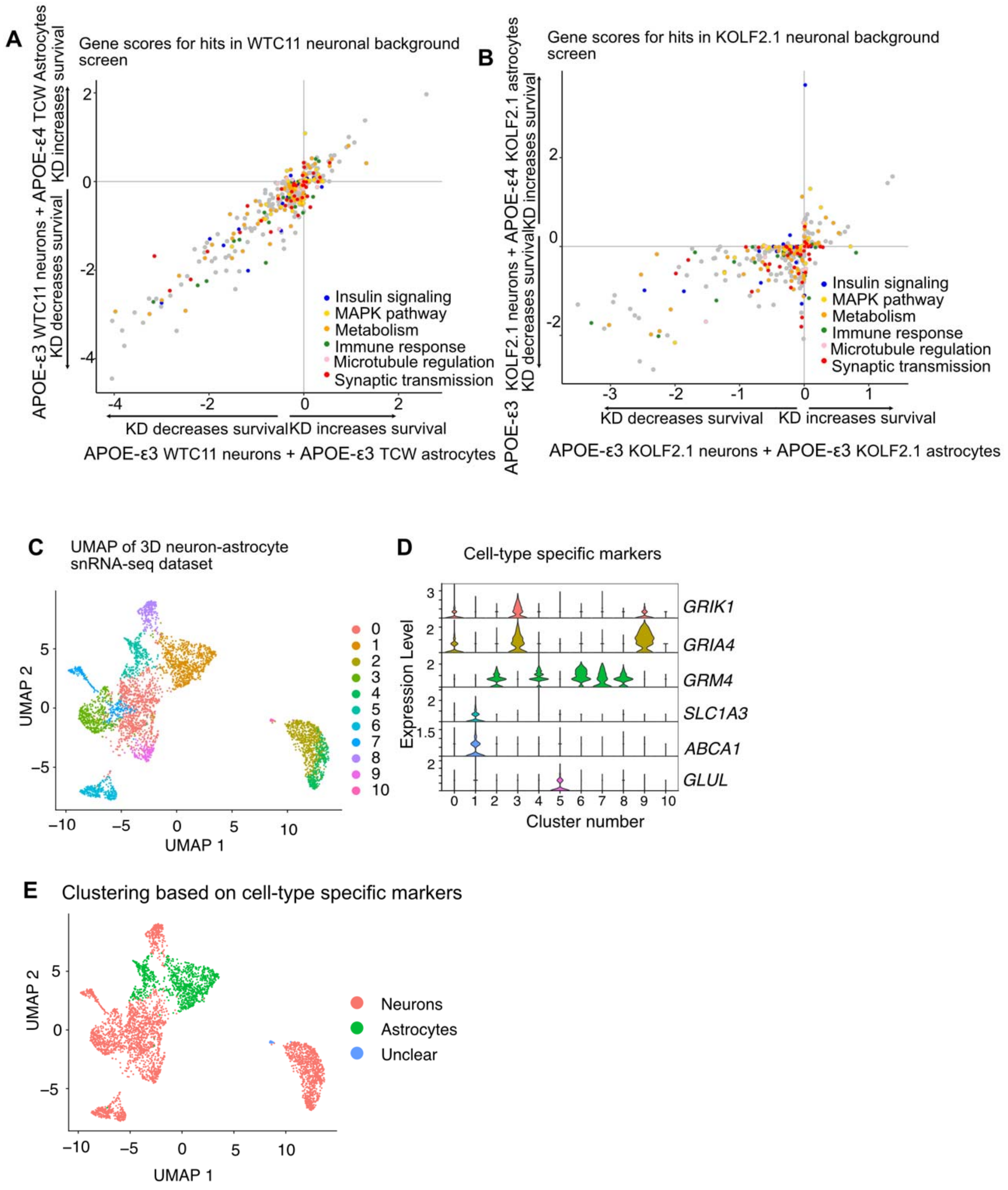


**Figure S7. CRISPRi-based screen for neuronal survival in APOE-ε3 versus APOE4-ε4 astrocyte 3D co-cultures and culture characterization** (related to Fig. 7)

**(A)** Scatterplot of gene scores from screens using APOE-ε3 versus  (x-axis) compared to APOE4-ε4 (y-axis) astrocytes (TCW 1E33-C, TCW 1E44-C) and APOE-ε3 neurons (WTC11). Hits (FDR < 0.05) from screens were included in the

bioRxiv preprint doi: https://doi.org/10.1101/2023.04.26.538498; this version posted August 12, 2024. The copyright holder for this preprint (which was not certified by peer review) is the author/funder, who has granted bioRxiv a license to display the preprint in perpetuity. It is made available under aCC-BY 4.0 International license.

scatterplot. Genes belonging to selected functional categories were annotated in different colors; other genes are shown in gray.

**(B)** Screen as described in B, but both neurons and astrocytes were generated in the KOLF2.1 background.

**(C)** UMAP representation of snRNA sequencing data of 3D astrocyte (APOE-ε3 and APOE-ε4) and neuron (APOE-ε3) co-cultures. Colors represent clusters assigned through unbiased clustering

**(D)** Cluster assignments based on expression of cell type-specific markers

**(E)** UMAP of final assignments based on cell type-specific markers. Neurons are highlighted in red and astrocytes are highlighted in green. Cells that have unclear assignments were removed
